# State of Mind Ireland-Higher Education: A Mixed-Methods Longitudinal Evaluation of a Positive Mental Health Intervention

**DOI:** 10.3390/ijerph17155530

**Published:** 2020-07-31

**Authors:** Niamh O’Brien, Martin Lawlor, Fiona Chambers, Wesley O’Brien

**Affiliations:** Sports Studies and Physical Education, School of Education, University College Cork, T12 YN60 Cork, Ireland; f.chambers@ucc.ie (F.C.); wesley.obrien@ucc.ie (W.O.B.)

**Keywords:** mental health, intervention studies, physical activity, psychological resilience, young adults

## Abstract

Objective: This study evaluates the impact of the State of Mind Ireland-Higher Education (SOMI-HE) Mental Fitness intervention on student wellbeing, resilience, and physical activity (PA) participation. Design: A mixed-methods research design, comprising of a self-report questionnaire, and semi-structured focus group interviews at pre, post and follow-up phases were employed. Participants were a sample of 134 higher education students (29% male: 71% female; mean age range 18 to 25 years old). The quantitative outcome measures of wellbeing, resilience and PA data were analysed using SPSS version 26.0, (IBM, Armonk, NY, USA) with appropriate statistical analysis. Qualitative data were analysed using thematic analysis to capture the long-term outcomes and impact of the intervention. Results: The results indicate a significant intervention effect on participants’ wellbeing (t (120) = −4.27, *p* < 0.001), PA levels (t (126) = 3.91, *p* < 0.001) and motivational readiness for exercise change (χ2 (1, *n* = 131) = 6.9, *p* < 0.009 (2–sided). Qualitative findings suggest a sustained long-term increase in PA and resilience skills for positive mental health, and reduced stigma and barriers to positive mental health. Conclusion: The findings demonstrate the effectiveness of the SOMI-HE evidence-based intervention, and beneficial outcomes of a salutary approach to higher education student mental health.

## 1. Introduction

Despite the rising mental health problems among students attending higher-level education in Ireland and abroad, young people remain a neglected population and are substantially overlooked from both a risk prevention and health promotion point of view [1,2,3,4]. The demands of change and adaption, specifically associated with opportunities and risk in attending higher education, is particularly recognised for making emerging adults vulnerable to too much stress, and as a result, depreciated health [5,6,7,8]. The years of attending higher education are a critical time to create a culture of positive mental health through intervening and preventing further consequences related to mental health problems [9]. Higher education institutes (HEIs) can holistically aim to see students thrive and reach their lifelong potential if they respond with positive mental health promotion to the well-established need for preventative and protective mental health intervention [2,5,10].

Positive mental health is seen as fundamental to human development, and to coping with adversity [11]. It is a science emphasising wellbeing indicators that assist individuals to feel and function well [12]. The terms positive mental health and mental wellbeing are frequently used interchangeably [13]. Promoting positive mental health or mental wellbeing at the societal, community and individual level involves building and supporting individual resilience, creating supportive environments and addressing the broader determinants of mental health [14,15]. What constitutes positive mental health is essentially the feeling of happiness that comes with psychological resources, including self-esteem, mastery and resilience to life’s stressors [15].

‘Mental fitness’ is a concept with strong connections to positive mental health, specifically as it reflects the teaching that we can enhance mental health through cognitive, behavioural and psychology education [16]. Mental fitness is associated with mental resilience [17]. Resilience can be described as one’s ‘ability to bounce back from stress’ [18] (p.166), while mental fitness can be defined as ‘the modifiable capacity to utilise resources and skills to flexibly adapt to challenges or advantages, enabling thriving’ [17] (p. 63). Mental fitness implies that similar to physical fitness, during our lifetime we can increase our brains fitness in strength, endurance, and flexibility through using valuable psychological resources and intentional activities that increase growth and development of the brain [19,20,21]. This is known as neuroplasticity, which describes the brain’s capacity for reshaping and ‘creating new neural connections and growing neurons in response to experience’ [22] (p. 34). The term ‘mental fitness’ is adopted by current intervention research, as it deemed approachable (less stigmatising), positive, and bolsters the idea that mental wellbeing is improvable [17,23].

The endeavour to establish effective positive mental health interventions to fuel resilience and enhance the lifelong quality of life is a purposeful investigation to safeguard the health of society [24,25]. Evidence-based positive mental health programmes in schools and colleges that aim to develop positive mental health or mental fitness skills in youth through using social and behavioural science theories of health behaviour are effective in positively impacting student wellbeing levels [26,27,28,29]. Effective positive mental health interventions generally include components such as mental health literacy [30,31], personal strengths [32], mindfulness [33,34,35], optimism [36], and goal setting [37,38]. Significant evidence also recognises the positive effect of using physical activity (PA) interventions as a means to enhance positive mental health [27,39,40].

It is well documented and clinically proven that regular physical activity (PA) participation enhances the quality of life, as it can reduce the risk of ill mental health and non-communicable diseases (NCDs) [41,42,43]. PA participation is also strongly associated with positive psychological wellbeing across all age groups and genders [44,45,46,47]. Furthermore, PA interventions are proven to enhance the mental health of various age groups and contexts [46,48,49,50]. There is significant evidence that recognises the positive effect of using PA interventions as a means to enhance positive mental health [27,39]. In a meta-analysis of psychological and exercise interventions, Barrantes-Brais et al., (2016) maintained that each type of intervention (positive mental health and PA) is similarly effective in impacting student wellbeing [27]. Emerging adults in Irish HEI’s (higher education institutes) who engage in regular bouts of PA participation are more likely to have higher perceived wellbeing when compared to physically inactive emerging adults [44,51]. Interventions designed to increase PA have significant outcomes among higher education populations when embedded within a university/college course, or designed using comprehensive models of behaviour change, such as the transtheoretical model [52,53,54]. Higher education is a pivotal period to engage young adults in healthy behaviours, particularly recognising the role of PA participation and wellbeing [44]. Regular PA participation, therefore, should be regarded as a viable tool for improving subjective well-being in emerging adults [55].

The challenge, however, with creating effective PA and positive mental health interventions is often the struggle to ensure that interventions are designed collaboratively, using conceptual frameworks, and supported by theory and evidence-informed methods to respond to the assessed needs of a target population [14,56,57,58]. At a structural level, this also means that addressing the personal, social and environmental determinants of risky, as well as health promoting behaviours, is essential for behaviour change and intervention design [59]. Dahlgren and Whitehead (1991) described a social ecological theory of health and referred to the determinants of health as ‘layers of influence’ [60]. The layered components of the social ecological model include personal (age, sex, hereditary), interpersonal (individual lifestyle factors), micro-environment (social and community networks and living conditions), and the broader socio-political environmental conditions (governing and policy). The Dahlgren and Whitehead (1991) framework has helped researchers and policymakers to construct a range of hypotheses about the social and environmental determinants of health. It acknowledges the interactions between the various determinants of health, allowing modifiable influences on health to be amended through social policy [60,61]. Many environmental determinants lie beyond the capacity of health interventions, as change often depends on the action taken by agents or groups across the layers of the environment [62]. However, Barry (2009) asserts that, at the individual level, psychosocial determinants of positive mental health can be addressed, and coping skills as well as protective mental health behaviours can be enhanced by interventions that promote cognitive and emotional resources, such as self-esteem, identity, self-efficacy, and resilience [14]. The SOMI-HE intervention specifically aims to address the determinants of positive mental health and PA at the individual level, as outlined by Barry (2009) [14]. This situates the current intervention primarily at the interpersonal level of the environment. The SOMI-HE intervention, however, also aims to operate across multiple levels of the environment by acknowledging that mental health is impacted through the interdependence of personal and broader social, economic and environmental determinants.

### 1.1. The Purpose of This Study

It is against the backdrop of the concern for low levels of wellbeing, resilience and PA among higher education students that the State of Mind Ireland-Higher Education (SOMI-HE) intervention was developed [51,55]. The present study aimed to evaluate the impact and effectiveness of the SOMI-HE Mental Fitness intervention in increasing wellbeing, resilience, PA level and motivational readiness for PA change. The specific objectives were to (1) investigate if student engagement with SOMI-HE can increase short-term indicators of subjective wellbeing, resilience and PA participation, (2) identify the demographic determinants (gender and age groups) of change in wellbeing, resilience ad PA over time, (3) establish the long-term outcomes of engaging with SOMI-HE.

### 1.2. The SOMI-HE Intervention Programme

The *SOMI-HE* programme [55] differs from *SOMI* [23]. SOMI-HE is designed specifically for higher education students and aims to (a) develop the knowledge and application of positive mental health strategies, (b) increase well-being and prevent ill mental health experiences through evidence-based practices, (c) reduce mental health stigma among higher education students and promote help seeking behaviour, and (d) to increase levels of physical activity according to the international guidelines. The previous SOMI intervention from Breslin et al., (2018) focused on increasing undergraduate athletes’ knowledge of mental health and engaging those with mental health problems and help-seeking intentions [23].

The SOMI-HE intervention programme was designed using a procedure known as intervention mapping (IM) [56]. Intervention mapping (IM) was proposed as a suitable systematic tool for developing innovative health promotion programmes for complex health problems through a collaborative, comprehensive theoretical approach [63,64,65,66]. The SOMI-HE is a two-part, three-hour-long intervention, pedagogically designed to engage a maximum of 150 students per sitting. A dosage of 2 × 90 min sessions across two weeks sees large cohorts of students participate in discussion, reflection, and positive mental health activities, through audio-visual presentations and engagement with an interactive student workbook—a take-home resource specifically designed to influence behaviour change in each of the intervention components (see Table 1). Examples of intervention behaviour change strategies include guided practice, elaboration, conscious raising, chunking of information, discussion, empathy training and modelling [29,67,68]. For a full description of the SOMI-HE intervention design procedure, content selection and delivery rollout, please refer to O’Brien et al., 2020 [55]. The current research tested the hypothesis that the SOMI-HE intervention (as designed using the IM framework) could increase perceived wellbeing, resilience, PA levels and motivation readiness for PA change.

## 2. Material and Methods

### 2.1. Participants and Setting

As part of this study, the principal investigators (PIs) used a mixed-method longitudinal intervention design, specifically to explore the impact of the SOMI-HE intervention programme on indicators of positive mental health. Mixed methods research is recommended for intervention researchers, as the research design combines approaches used to strengthen intervention design and implementation [69]. Several reasons outlined by Meissner et al. (2011) underline the adoption of mixed methods in this SOMI-HE research—the authors maintain that mixed methods allow the researcher to look at a problem from various perspectives, contextualise the setting, and validate or compare results [70]. It was essential in the process of the intervention design and evaluation to listen to the voices of the stakeholders and participants.

Additionally, qualitative components of research add depth and meaning to empirical findings, which can assist in assessing the feasibility and the potential for intervention, through gathering preliminary evidence and informing researchers of the barriers to effective intervention design and adaption [69] (p. 27). Conversely, quantitative methods can identify patterns and trends [69]. Using these combined research methods can enhance knowledge of the outcomes of the programme, and can inform the development of future research as they are considered a ‘systematic and rigorous form of inquiry’ [70].

Eligibility criteria included all students attending higher education over the age of 18 years. A clustered, convenience sample of two mixed-gender student cohorts from the same degree programme completed the SOMI-HE study over two academic years. The participants in this study were allocated discretionary time from their formal timetable to complete the SOMI-HE intervention programme. Access to students was made available by a ‘champion’ of the research in the host institution. The intervention was delivered by the lead researcher. The lead researcher was formally introduced to the participants as a research student and had no previous association with the participants of the intervention in any capacity.

**Figure 1:**
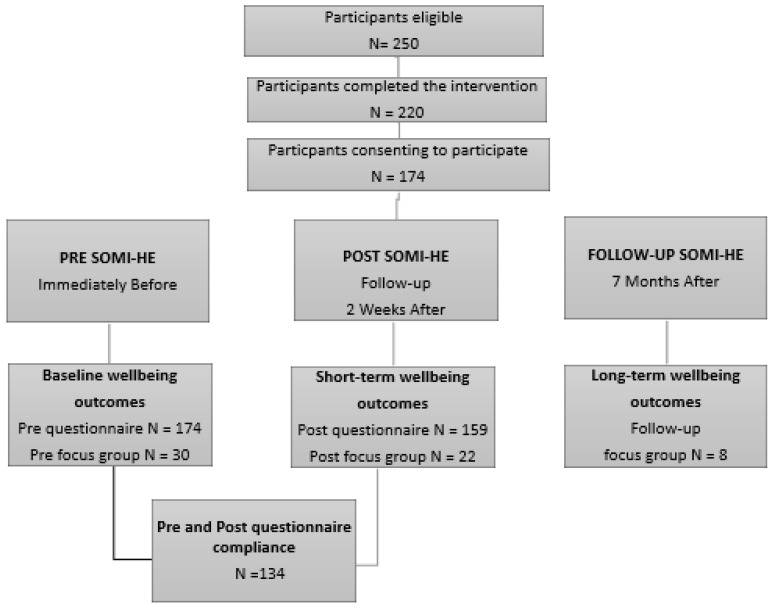
Consolidated Standards of Reporting Trials (CONSORT) diagram of data collection at pre, post and follow-up intervention stage.

The SOMI-HE study was implemented twice—in February 2018 and 2019. Each time the intervention was delivered, data were collected across three-time points, specifically to assess the short term and long term wellbeing outcomes at baseline (pre-intervention), post-intervention (2 weeks) and at follow-up phase (7 months after intervention completion) (see Figure 1). A total of 250 participants were eligible and informed about the SOMI-HE from a programme content, scheduling, research, and ethical consent perspective. Overall, 88% (*n* = 220) of the sample attended the programme, while 70% (*n* = 174) of the sample provided consent for completing the research element to the study. Out of the 174 consenting participants, 134 were included in the study (29% male and 71% female). Seventy-five per cent of participants were aged between 18 and 25, 14% were aged between 26 and 29, and the remaining 11% of the sample was 30 years and over. The majority of the students (84%) were in their first year of a postgraduate education programme.

### 2.2. Ethical Considerations

The University College Cork Social Research and Ethics Committee reviewed and approved this study (Log No 2017-009) in March, 2017. Prior to participation in SOMI-HE, each participant was supplied with an information sheet, and written consent was a pre-requisite prior to the completion of specific data measurements. All participants invited to partake in the research were informed of their right to withdraw from the proceedings at any stage. In the situation where a student became distressed by the survey, interview or programme content, internal and external university support service information was provided. For example, each student’s consent form at the beginning of data collection provided such support service information, and these contact points were also reinforced on the student take-home workbook, and at the end of the intervention. Students were aware that the research was an optional component for completing the SOMI-HE intervention programme.

Researcher bias can be referred to as a distortion of results influenced by the researcher’s values or the interest of research funding bodies [71]. Ethical awareness is the undercurrent of this study. The research objective is to create evidence-based interventions that are aimed to work effectively in preventing ill mental health through the promotion of positive mental health. If the research programme was deemed ineffective, it is evidence that such a strategy is not helpful to the cause and is therefore still a reported outcome. The lead researcher took measures to continually demonstrate reflexivity and prevent such bias through external auditing, peer consulting negative case analysis, and triangulating the results [72].

### 2.3. Study Design and Procedures

Qualitative and quantitative approaches are necessary to give depth and breadth of research in social and behavioural sciences [73]. Interventionists looking to develop evidence-based practice can enhance intervention design and research rigour through the use of mixed-method research design [69]. A wellbeing questionnaire, comprising several empirically valid and reliable psychometric and physical activity scales, and repeated semi-structured focus group discussions were utilised for pre and post-intervention data collection time points, while semi-structured focus group discussions only were used for the follow-up stage of data collection.

### 2.4. Quantitative Participants and Recruitment

A self-report wellbeing outcomes questionnaire was utilised to obtain quantitative data, specifically as it provides large amounts of data unattainable by way of qualitative methods, is easily analysed and regarded as unbiased due to the standardised parameters for reliability and validity [74]. Approximately 20 min prior to the intervention rollout, the students had the option to complete the survey online using SurveyMonkey^™^, via a link or hard copy distributed by the research team. The survey consisted of several standardised questionnaire instruments.

### 2.5. Qualitative Participants and Recruitment

**Figure 2:**
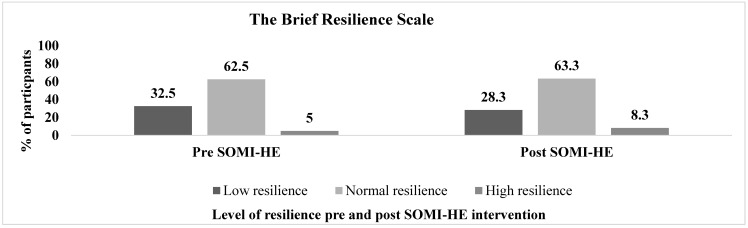
Levels of resilience pre- and post-SOMI-HE intervention.

During the introduction of SOMI-HE (two weeks in advance of its delivery), focus group volunteers were requested to email the researcher. Thirty-two students contacted the leading researcher, expressing interest for focus group participation within 24 h. A total of 30 participants completed the pre-intervention semi-structured focus groups interviews, while 22 participants completed the post-intervention semi-structured focus group interviews. At 7 months follow up, the research team retained 8 participants from the original pre/post sample for the semi-semi structured focus group interview (see Figure 2). The focus group discussions were composed of 21 female and 9 male participants at pre, 15 female and 7 males at post, and 6 female and 2 males at follow-up.

### 2.6. Measurements

Self-report wellbeing outcomes questionnaire: participant demographic characteristics were obtained and measured to determine the impact of the programme on the following variables: wellbeing, resilience, and PA participation.

#### 2.6.1. The Warwick-Edinburgh Mental Wellbeing Scale (WEMWBS)

The WEMWBS was developed to enable the monitoring of mental wellbeing across the UK’s general population [75]. The WEMWBS is a 14-item scale validated with student and adult populations and stands as a robust tool, with excellent test-retest reliability intraclass correlation coefficients (ICC) at 0.83 [13,51]. The scale has been recommended for the use in evaluating mental health promotion initiatives and programmes at both a group and individuals level [75,76]. The results of the WEMWBS are presented as mean scores in the descriptive analysis only, as the WEMWBS was not designed to screen cut-off scores of wellbeing [77].

#### 2.6.2. The Brief Resilience Scale (BRS)

The BRS measures resilience or ‘the ability to bounce back from adversity’. This instrument consists of six items with a higher score indicating higher levels of resilience. The BRS has demonstrated satisfactory internal consistency and test-retest reliability (ICC.69). The scale has also been used in the context of a university setting in previous research, and has proven valid and reliable when examining resilience levels among higher education students [18,78]. The score of the BRS can be categorised. A score between 1 and 2.99 represents low resilience, a score of 3.00 to 4.30 represents normal resilience, and scores between 4.31 and 6.00 represent high resilience.

#### 2.6.3. The Patient-Centred Assessment and Counselling for Exercise Plus Nutrition (PACE+ Physical Activity Measure)

The PACE+ is a two-item screening tool used among adolescents in primary care. Participants (over 18 years) are asked: ‘Over the past 7 days, on how many days were you physically active for a total of at least 30 min per day?’ and ‘Over a typical or usual week, on how many days are you physically active for a total of at least 30 min per day?’. Responses are numbered 0–7 days. A composite average of the 2 items yields a score of days per week the participants can accumulate 30 min of MVPA. A binary score can be created using PACE+ with five or more days per week categorised as meeting the recommended PA guidelines for health. The PACE+ demonstrates excellent test-retest reliability across multiple groups and subgroups of adolescents (ICC 0.77) [79]. In studies assessing the reliability of the PACE+ in higher education student populations in Ireland, the results indicate that the PACE+ has strong test-retest reliability (ICC 0.70), and shows high accuracy of those not meeting the PA guidelines (73.5%) [80].

#### 2.6.4. The Physical Activity Stages of Change Questionnaire (PASCQ) or Transtheoretical Model (TTM)

The PASCQ was designed to assess the stages of motivational readiness for change mode in individuals as they move through a series of stages on the transtheoretical model (TTM). The TTM is an integrative, biopsychosocial model that seeks to conceptualize the processes of intentional behaviour change that include five distinct stages: pre-contemplation, contemplation, preparation, action, and maintenance [82]. The PASCQ is a four-item binary questionnaire with a ‘yes’ or ‘no’ response scored by an algorithm. For example, if an individual’s response to question one (‘I am currently physically active’) is ‘no’, and question two (‘I intend to become more physically active in the next six months’) is ‘no’, they are identified as being in the pre-contemplation stage on the TTM. By matching where an individual lies on the stage of change, an appropriate intervention strategy can be implemented [81]. The PASCQ demonstrates very good test-retest reliability (ICC 0.85) [83]. The validity of the instrument was determined as satisfactory when compared to other direct measurements of PA (accelerometer, maximum oxygen consumption VO_2_ etc.) [84,85]. The PASCQ has been used among the college student population in recent years [85].

#### 2.6.5. Focus Group Discussions

Semi-structured focus group discussions followed the recommended programme evaluation guidelines, as outlined by Krueger and Casey (2015) [86]. Focus group discussions were conducted using repeated questions at pre, post and follow-up intervention stages. This method aimed to capture and monitor the outcomes of the programme based on the expressions of the returning participants. Ten questions were devised, piloted, and verified by other members of the research team. Research questions 1–5 investigated students’ knowledge, skills and attitude towards positive mental health and strategies. Questions 6–9 sought to understand student PA levels, behaviours, and attitudes. Question 10 asked participants what they hope to gain (at pre) and what they had gained from the intervention programme (at post and follow-up).

All focus group discussions were audio-recorded via a digital dictaphone (Olympus Digital Voice Recorder WS-852), transcribed verbatim and anonymised. The leading researcher, four trained interviewers and five assistant moderators facilitated and took notes throughout each discussion. Assistant moderators were consulted proceeding each interview on shared insights and interpretations of the participant discussions. The process of data analysis was verified by a qualitative research consultant within the university.

## 3. Data Analysis 

### 3.1. Self-Report Questionnaire

Only participants who completed both pre- and post-intervention self-report questionnaires were included in the study, and where any of the questionnaire scale data were incomplete, the responses were excluded from that specific scale analysis. In a case by case analysis of extreme values, using detrended normal Q-Q plots and box plots for each scale, if data appeared inconsistent or invalid, outliers were removed. This led to a reduction in sample size; however, contaminant observations may affect the bias of the statistical result in various ways [87]. In intervention studies, 70 participant cases provide adequate power to detect a moderate effect for an intervention [88]. The Cronbach’s alpha coefficient indicated good internal consistency for the WEMWBS (α = 0.88), the BRS (α = 0.84), the PACE+ (α = 0.87) and the PASCQ (α = 1.0). In each of the scales used, the score did not appear to suffer from floor and ceiling effects in either sample.

Several statistical tests were used to interpret the data collected, using Statistical Package for the Social Sciences (SPSS), Version 26.0 (IBM, Armonk, NY, USA) for Windows. The WEMWBS, BRS, and PACE+ were totalled using the mean score and standard deviation calculations. A mean cannot be interpreted for the PASCQ, as it is a binary type questionnaire. Levels of resilience and PA were computed and assigned to subgroups at both pre- and post-intervention data collection points. A binary variable was created for the PACE+ and the PASCQ scale items. Specifically, levels of PA using the PACE+ were categorised into (1) those meeting the recommended PA guidelines and (2) those who were not meeting the recommended PA guidelines. Using the PASCQ stages on the TTM, data were divided into either those on the (1) lower (pre-contemplation, contemplation, preparation) or (2) upper stages (action and maintenance) of exercise readiness. To address the previously identified research questions, differences in pre- and post-intervention scores for wellbeing, resilience and PA were calculated using a combination of t-tests, McNamer’s test and repeated measures ANOVA. Gender differences in wellbeing, resilience and PA at pre-intervention were investigated using independent sample t-tests. Paired sample t-tests investigate whether there were changes in mean levels of wellbeing, resilience, and PA at pre- and post-intervention. McNemar’s tests were used to identify the changes in the binary variables created in the PACE+ and the PASCQ. Individual repeated measures ANOVA were conducted to explore the changes in wellbeing, resilience, and PA using controlled demographic determinants including gender (male or female) and age (18–25 years or 26 years plus). Statistical significance for all tests was set at *p* < 0.05.

### 3.2. Focus Groups

Using a thematic analysis approach, outlined by Braun and Clarke (2013) [89], the data were analysed through reading, re-reading, generating codes, searching for themes, defining and naming themes, and reporting. The questions were presented through a deductive approach and interpreted through an inductive approach lens. All data were extracted from the pre-existing coding frame that was shaped by the research questions. Following the procedure provided by Bree and Gallagher (2016) [90], themes were initially processed using Microsoft excel. Bree and Gallagher (2016) [90] provided researchers with a physical process of managing data analysis that reflects the thematic analysis framework, as described by Braun and Clarke (2013) [89]. The process is considered a scientific tool to assist the thematic analysis and triangulation of qualitative data, collected by organising, coding and classifying data through using the colour and sorting features of Microsoft Excel [91]. Using a thematic map, themes were matched across three-time points (pre, post and follow-up), and commonalities/differences across each of the focus group discussions were integrated and reported using a contiguous approach [92]. Thematic analysis was used to evaluate the core themes expressed by the participants overall, followed by a convergence triangulation process, specifically to verify and validate the quantitative data [93].

## 4. Results

### 4.1. Self-Report Questionnaire Findings

#### 4.1.1. Descriptive Results

Study participants included one hundred and thirty-four higher education students, who were matched as part of the pre- and post-intervention data collected. Table 2 provides an overview of the demographic characteristics of the SOMI-HE study sample. Table 3 presents the mean scores for the each of the scales, as differentiated by gender, at both the pre- and post-intervention data collection stages of SOMI-HE.

**Figure 3:**
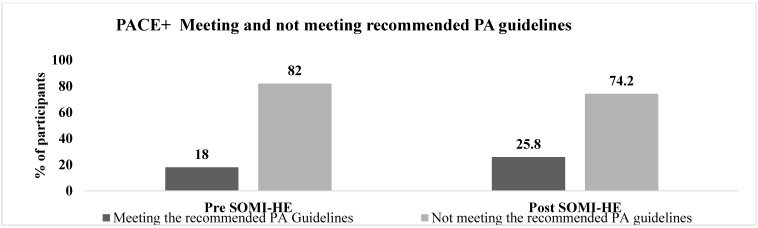
Percentage of students meeting/not meeting the recommended physical activity (PA) guidelines pre and post SOMI-HE intervention.

**Figure 4:**
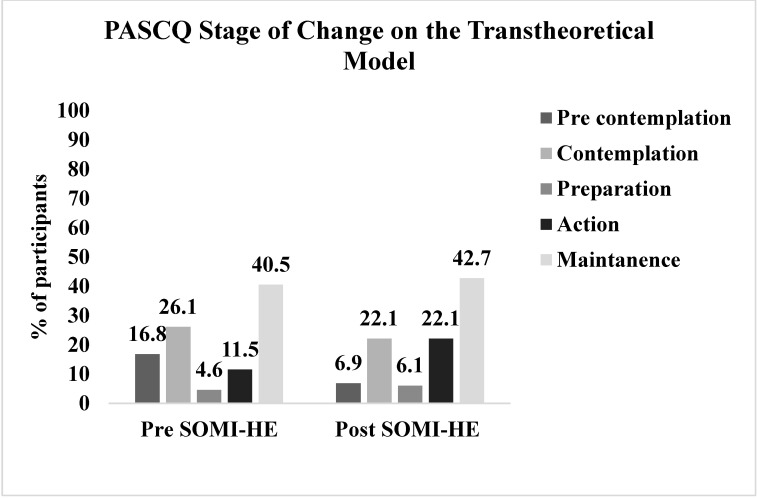
Percentage of students on each stage of the transtheoretical model (TTM) pre- and post-SOMI-HE intervention.

Figure 2, Figure 3 and Figure 4 illustrate the pre- and post-intervention descriptive data for the BRS, PACE+ and PASCQ instruments, according to the data’s categorical cut-off points. 

#### 4.1.2. Wellbeing, Resilience, PA, and the SOMI-HE Intervention 

A paired sample t-test was conducted to evaluate the impact of the SOMI-HE intervention on participants’ perception of their wellbeing, resilience, and PA. There was a statistically significant increase in wellbeing scores using the WEMWBS from pre (M = 44.52 ± 7.53) to post (M = 48.28 ± 7.52), t (120) = −5.27, *p* < 0.001 (two-tailed). The mean increase in wellbeing scores using the WEMWBS was 3.76, with a 95% confidence interval ranging from −5.17 to 2.35. The BRS did not indicate a change over time (see Table 2). In relation to PA levels, the PACE+ results suggest that there is a statistically significant change in PA level between pre (M = 2.96 ± 1.6) and post (M = 3.5 ± 1.70), t (126) = 3.91, *p* < 0.001 (two-tailed). In all instances, the eta squared statistic (50) indicated a large effect size. A McNemar’s test used to measure whether the intervention had a significant impact on increasing the number of participants meeting the recommended PA guidelines indicated that there was no significant change over time (χ2 (1, *n* = 131) = 2.72, *p* < 0.096) (2–sided)). To interpret the effect on participants’ TTM stage of change toward PA participation, an additional McNemar’s test showed that the two levels of lower and upper stages of exercise readiness within the TTM were different over time, (χ2 (1, *n* = 131) = 6.9, *p* < 0.009 (2–sided)), with a 13% increase in participants moving from the lower stages at pre-intervention to the upper stages of exercise readiness on the TTM at post-intervention.

#### 4.1.3. Gender Differences Wellbeing, Resilience, and PA at Pre-Intervention

An independent sample t-test was conducted to compare the mean wellbeing, resilience, and PA self-reported scores differentiated by gender at pre-intervention. A significant difference in the mean wellbeing scores for males (M = 47.205, SD = 7.21) and females (M = 43.47, SD = 7.44) using the WEMWBS (t (119) = 2.503, *p* = 0.014) was revealed. Similarly, a significant difference in the mean resilience scores for males (M = 3.47, SD = 0.58) and females (M = 3.05, SD = 0.69) the BRS (t (118) = 3.096, *p* = 0.002) was identified. There was no significant difference in the mean PA self-reported scores using the PACE+ (t (124) = 1.317, *p* = 0.190).

#### 4.1.4. Demographic Determinants (Gender and Age Groups) of Changes in Wellbeing, Resilience Ad PA Over Time

A series of 2 × 2 independent repeated measures ANOVA were conducted using the WEMWBS, the BRS and PACE+ to investigate if wellbeing, resilience and PA scores differ between the demographic determinants of gender and age (between-subject factors) at the pre- and post-intervention stages. For each of the repeated measures ANOVA’s, Mauchly’s test indicated that the assumption of sphericity had been violated, *X²* (0.0) = 0.000, *p* = 0.0. Therefore, degrees of freedom were corrected using Greenhouse–Geisser estimates of sphericity (ε = 1.0). Among both of the demographic determinants for gender and age, the results show there were no significant differences to wellbeing over time in gender (F (1.0, 119.0) = 2.32, *p* = 0.130) or age (F (1.0, 119.0) = 0.051, *p* = 0.821). In relation to resilience, the results show that there was a statistically significant effect for time on gender, with females showing a greater increase in resilience levels (F (1.0, 118) = 0.59, *p* = 0.045), while there no significant increase in resilience among the age groups (F (1.0, 118.0) = 0.023, *p* = 0.879). There was no significant effect of time on gender for PA levels at pre- and post-intervention stages (F (1.0, 113) = 1.50, *p* = 0.224) or age group F (1.0, 124.0 = 0.010, *p* = 0.920).

### 4.2. Focus Group Findings

Five prominent core themes emerged from the qualitative data across each of the three time- points, which indicated that higher education settings are an essential environment for positive mental health promotion, specifically in responding to the needs of the young adult population.

(1) Stress stigma—a barrier broken by peer connection. At pre-intervention, participants expressed concern for the mental wellbeing of their peers, reporting that they observe high levels of stress and anxiety among their age group. Participants were worried about their low levels of mental health literacy, and therefore, felt burdened with concern for their efficacy towards helping someone else. One female postgraduate participant asserted: ‘You can see in the course that there are lots of people who are struggling…’. At the post-intervention stage, the reflections made by the participants turn more inward. The participants discuss how the programme addressed the stigma related to personal stress and discussed the benefits of knowing that ‘everyone has mental health’. Participants moved towards feeling less isolated in experiencing high levels of stress at post-intervention. When discussing the activities in SOMI-HE, a female postgraduate reflected: ‘You can feel a little bit isolated I think, you’ve got so much to do. It was really nice to see that everyone is kind of dealing with the same issues.’ At the follow-up phase, participants recalled the relief of stigma associated with stress and discussed how the programme addressed their understanding of stress. Participants highlighted again that they had felt isolated, and once they spoke to their peers through the medium of the SOMI-HE intervention, they felt connected. Participants observed that you cannot see the signs of stress or poor mental health in yourself or others if you are not connecting. This was particularly evident in an exchange between a female and male peer at follow-up: The young female expressed: ‘I thought I was the only one to feel anxious and stuff, now it’s like wait, there are so many more people that feel that same way as me. I can actually open up a bit here now.’ Her male peer agreed: ‘You can actually... I even find this year in the course I do tell people I feel a bit anxious and I don’t feel…I’m not ashamed of it.’

(2) The perceived barriers to positive mental health. At pre-intervention, participants frequently discussed barriers that include concepts such as mental health literacy, stigma towards mental illness, resilience skills, time, personal wellbeing negligence and academic pressure. At post-intervention, participants responded to a discussion on the programme outcomes. The barriers addressed included increased mental health literacy, reduced mental health stigma, enhanced resilience-building skills and self-awareness of their personal mental health or wellbeing. In a discussion on stigma, one participant summarises the type of barriers addressed by the intervention.

‘…mental health courses or wellbeing courses in today’s world, they’re all kind of focussed on speaking up and ignoring the stigma. I found this course actually gave you techniques of what you can actually do besides just talking out about it, and it actually showed you how to manage and what you should actually do besides just having the confidence to speak about it in the first place.’

The SOMI-HE programme activities provided participants with efficacy and skills to overcome their perceived barriers to positive mental health. One female participant reported: ‘the programme helped me identify my own capacity and resilience… it helped me think about what I was going to do to get that stress level down’. At follow-up, the long-term perceived barriers addressed by the programme again included mental health literacy, stigma, resilience skills, self-awareness, and peer connection. Participants expressed having enhanced efficacy to make positive decisions for their wellbeing and how to respond to the wellbeing needs of others. One male postgraduate participant discussed how modelling compassion and promoting positive mental health can help individuals and the broader community, asserting:

‘I think it spreads around, say like the community, even if I was talking to one of the lads it would be “are you alright?” or “do you need a hand with this?” or whatever. So, it definitely feeds into other people if you start doing it. It’s a knock-on effect basically.’

(3) Help-seeking—A determinant not addressed by the SOMI-HE programme. In the original SOMI-HE programme design, help-seeking was understood as a determinant of positive mental health. SOMI-HE, therefore, had signposting components that directed participating students to college support services. At the pre-intervention stage, participants frequently discussed the burden of not being able to help their peers with mental health problems. At post-intervention, participants conveyed a continued lack of knowledge on the types of mental health supports, therapies, treatments, and their costs. Follow-up data reiterated this, with participants articulating they had no road map to follow in the event of needing to help others. For one participant, the absence of knowledge of how to support her peer outside of college dismayed her, as she revealed:

(4) A realization—exercise is good for you. During the pre-SOMI-HE discussion, PA emerges as the most commonly used method of stress management. Participants often reported being more physically active prior to attending college. Participants maintained that the barriers that prevented them from increasing their PA levels included effective time management, poor mental states, tiredness, and third-level academic workload challenges. A participant who had previously studied nutrition expressed: ‘diet and exercise would be a big part of my life, but I do think your academic work will suffer…it’s really hard to try to find the balance’. At post-intervention, participants communicated a change in efficacy toward their ability to be more physically active while at college. Participants made statements such as: ‘You have to actually make time and to take responsibility and ownership’. At follow-up, the participants reported a lasting influence from the programme on their PA levels and efficacy toward increasing their PA. They discuss a positive effect from engaging in PA and reflected on the increased awareness of the benefits of PA for positive mental health. One male participant reflected:

‘I made more of an effort during the summer. Knowing how it (PA) affects your mental health, knowing that it will benefit you and you won’t regret it…it’s that realisation I suppose that only good things can come from it.’

Another female peer described recognising that PA is a tool to help her cope with stress, disclosing:

‘I think the whole exercise element really kind of hit me as well… I kind of realised that, no, I have to exercise to think clearly and all that… I thought I had to run away from my problems, I actually have to go for a run to actually face them.’

(5) The gap is essentially being provided with an opportunity to learn. It was identified at the pre-stage of data collection that participants felt they needed more support from higher education institutions to cope with the challenges and changes that come with attending college, upholding they ‘don’t think enough is being done.’ Many participants identified that they needed to learn skills as they do not feel they adequately look after their mental health. A female undergraduate participant disclosed:

‘I find it extremely difficult to juggle everything together. If I was given the tools and maybe told maybe how I could do my work and then still have a social life and still be happy… I do get very, very stressed I must say. Before coming here, before this course, I used not to get stressed. I’d find myself getting very anxious I have to say.’

During post-intervention data collection, participants reflected that not only did they report learning new skills, strategies and efficacy to discuss mental health, they also reported an increase in awareness of how they were not looking after their mental health previously. Participants identified that maintaining wellbeing and building resilience was something that you must work on. A male participant stated while reflecting on his mental health awareness that ‘…you can lose track of sight, and you might lose focus… I just learned from it that you need to take a step back and think about yourself as well.’ Follow-up focus group discussions indicate there is a lasting positive effect from exposure to a positive mental health intervention, such as the SOMI-HE. Participants describe an increase in self-awareness and positive mental health strategies, including mindfulness, meditation, PA, connecting with others and using positive affirmations. One female postgraduate participant voiced her efficacy to maintain her wellbeing stating: ‘I genuinely came out feeling ya, I’ve got this’.

## 5. Discussion

This study aimed to examine the outcome of an evidence-based behaviour change intervention programme designed to promote the wellbeing, resilience, and PA levels of higher education students. It has been well documented that low levels of PA participation and poor mental health are an increasing concern among the emerging adult population attending higher education [1,5,51,94]. The need to promote positive health through intervention in higher education settings has been recognised as a viable and effective method to address the health concerns of this age group [5,6,26,27,95]. Evidence-based interventions designed using the IM protocol from Bartholomew Eldridge et al., (2016) have been recognised for assisting health promotors in developing the best possible intervention [55]. Evidence emerging from the quantitative statistical and the qualitative focus groups findings in the current research suggest that the SOMI-HE intervention engenders short-term and long-term effects within the variables of positive mental health.

Through combining both qualitative and quantitative data methods, the converging data results indicate complimentary positive mental health outcomes. Participants reported an increase in perceived levels of wellbeing and a reduction in barriers over time towards positive mental health. Barriers such as mental health literacy, stigma, resilience skills and self-awareness of personal mental health were identified as positive mental health obstacles reduced through engaging with SOMI-HE. These identified barriers are interrelated, and when weaved together, they highlight the co-dependency of intervention components, such as stigma, mental health literacy, resilience and wellbeing, similar to other intervention research [96,97]. In other Irish research, higher education students report that young people want informal mental health-promoting techniques and opportunities to talk about improving their mental health knowledge [98]. It seems plausible to assume that SOMI-HE is an effective medium that gives HE students that opportunity for developing their understanding of wellbeing and mental health knowledge, as it provides participants with a necessary platform for learning about mental health and resilience skills. Mental health systems have become imbalanced, with an overreliance on the treatment of mental health, causing the ‘prevention gap’ [99,100]. SOMI-HE proposes as a method to close that ‘gap’ through using positive mental health strategies to reduce the incidence and prevalence of mental illness over the life span [101].

Addressing stigma, and particularly ‘stress stigma’, was positively associated with positive mental health, as participants described how discussing mental health with peers helped them to feel less isolated and more connected. Social relationships are essential to promote wellbeing and act as a buffer against ill mental health [12]. This finding emphasises how social connection was promoted through the intervention and gives prominence to the idea that individuals do not adapt to stress in isolation, instead they need a community when learning to build resilience to stress [102]. The issue of limited connection with peers reiterates how the environmental change that comes with attending higher education does not always lend itself to providing students with the essential social support systems and personal resources that contribute to building resilience and maintaining wellbeing. Therefore, to effectively increase wellbeing while attending higher level education, it is essential to acknowledge and evaluate the importance of the broader social and environmental layers that determine positive mental health [103]. The SOMI-HE intervention was designed to primarily address interpersonal determinants of mental health; however, due to the interconnectedness of the layered components of the social ecological model, it would appear that their engagement with the programme was influenced at the micro layers of the environment through enabling social connection. Participants upon completion of the programme appreciated the occasion and embraced the intervention as a time to discuss mental health with peers. This accentuates the importance of having the capacity to address the broader social and environmental determinants of mental health for dynamic intervention design. For example, González-Zamar et al., (2020) argued that the impact of the learning environment and practical applications of learning in higher education can either benefit or impede upon students’ sense of belonging and wellbeing [104]. Therefore, to adequately enhance social wellbeing in higher education, organisations need to consider the impact of the educational space and learning methodologies that influence students’ communication and interpersonal relationships. The programme, however, had little or no impact on the broader social and environmental determinants of mental health, as the implementation of the programme was subject to the permission of an agent in the broader layers of the student’s environment. This emphasises the role of pursuing collaboration and consensus within an institution for meaningful change and indicates that to truly maximise the outcomes of positive mental health interventions, they must be delivered and recognised at a structural level or a whole setting approach, as endorsed by other experts in the field [2,26,105]. Inaccessibility to influence the broader layers of the programme are deemed a weakness of the intervention study. A strategic lead responsible for implementing effective interventions is necessary for successful government and policy level ‘buy-in’ within the university context.

Diverging results indicate there was no significant impact on students’ perceived level of resilience (the ability to bounce back from adversity) using the BRS [18], while contradictory focus group findings identify an increase in resilience building skills, at both short and long-term phases. Several factors need to be considered to explain these findings. Firstly, it could be argued that time is a typically influential factor in acquiring any new skill. Consequently, it is challenging to measure outcome resilience levels as part of a short-term data collection strategy [106]. This consideration has also been found in previous studies [23]. However, in this instance, it could also be a question of accuracy of what we are measuring. Resilience can be conceptualised and measured as either an outcome in the face of adversity or a process of cognitive and emotional reactions and behaviours that facilitate both resistance to and recovery from stress [102]. It is possible that the BRS outcome-oriented measurement of resilience does not reflect the underlying assumption of the SOMI-HE intervention that positive mental health can be improved and that mental fitness can be developed to achieve further growth and optimal functioning [17]. After all, building resilience is considered a dynamic biopsychosocial process [107] Therefore, to reduce the measure of resilience to a single outcome of resilience level rather than resilience processes fails to recognise the broader factors associated with resilience. An individual’s level of resilience is circumstantial and determined by the available resources and skills that drive them to learn, grow and adapt to their experiences and environment [17,107]. Therefore, resilience cannot be measured in isolation—it needs to be put into action with the focus not on resilience outcome level per se, but on the capabilities and resources that are associated with the outcome of resilience [108]. It is advantageous to accurately assess resilience intervention studies, by employing a combination of measures of resilience to ‘provide clarity regarding which facets of resilience are related to psychological health and are most sensitive to change’ [106] (p. 7).

The association between mental health help seeking and mental health literacy has been identified in previous studies [109,110]. Teaching students about the benefits of mental health treatment may be an effective strategy to increase the prevalence of help-seeking behaviours [9]. Additionally, studies have suggested that interventions that address mental health literacy also need to address stigma, rather than simply educating about depression symptoms [97]. Stigma is a particular component of the SOMI-HE programme that was effectively influenced by the intervention design process. However, despite these positive findings, help-seeking was identified as an unsuccessful outcome of the programme. It emerges that sign-posting components of SOMI-HE (such as consulting your GP) that ought to enable effective help-seeking were identified as an unsuccessful outcome of the programme. The results suggest that the programme did not give students a recovery ‘road map’ towards the various types of mental health professional support options, and costs outside of college. Students expressed a continued burden and dismay to adequately guide their peers to mental health recovery services. This finding is most likely an issue that can be identified as a shortcoming of mental health service provision that is much larger than that of the SOMI-HE intervention. Despite advances in the youth mental health services in Ireland, a structure that enables swift access to services and supports is still lacking [111]. These finding in the SOMI-HE research are in line with the first principal of the recent Global Framework for Youth Mental Health which emphasises the need for rapid, easy and affordable access to care to ensure that a young person in need of youth mental health services can gain access to a service without referral or ‘gatekeepers’ to cross [112]. In Ireland, the journey has begun to remove GPs and emergency departments as the gateway entry to mental health care. McMahon et al., (2019) acknowledged that mental health services and supports in Ireland remain arduous; however, they proposed that in the next stage of development in youth mental care and support, a single free, confidential access point is needed to provide rapid and appropriate signposting for routine, urgent and emergency referrals [113]. Until this time arrives, this is a concerning misalignment between the promoting help-seeking components of the SOMI-HE intervention and the actual availability of access to help.

Although the SOMI-HE programme did not significantly increase participants level of PA to the point of reaching the recommended weekly guidelines (150 min per week), there was a significant reported rise in PA minutes per week. Small changes in PA (even less than half of the current recommended 150 min per week of MVPA) can lead to marked and clinically relevant changes in health status (particularly in inactive populations) [114]. Additionally, a significant proportion of participants moved from the lower stages of the TTM at pre-intervention to the upper stages of the TTM at post-intervention, suggesting through using a multi-theory approach outlined by IM that the SOMI-HE intervention impacted participants’ motivational readiness for change in PA effectively [55,115]. When merging these PA findings with the post-intervention qualitative data, there was a positive change in student efficacy toward increasing their PA levels. Specifically, participants transitioned from framing time as a barrier for PA participation (pre-intervention) to acknowledging that time barriers can be reduced by deliberately allocating the time to take part in PA. Self-efficacy found through PA interventions using the TTM to explain individual behaviours has been identified as a factor that contributes to the transition from the preparation stage to the action stage of the TTM among university students [116]. This reflects that the behaviour models used to inform the practical strategies in SOMI-HE were used effectively. Follow-up findings showed a continued long-term awareness and positive effect from engagement in PA as PA was conveyed as a mechanism to cope with life’s stressors. Both psychological and exercise interventions show efficacy in improving wellbeing in college population studies [27]. It is plausible to assume that an evidence-based intervention, such as SOMI-HE, designed by combining these two mechanisms (positive mental health and PA), is a complementary approach to increase third level student wellbeing.

It would appear from the quantitative findings that the female proportion of the sample reported the lowest levels of wellbeing and resilience at pre-intervention. Similarly, other Irish research indicated that female university students report lower levels of wellbeing and resilience than males [98,117]. When investigating the demographic determinants (gender and age) contributing to change in wellbeing, resilience and PA over time, it would appear from the quantitative findings that the female proportion of the sample reported the most significant increase in resilience only. Gender specific effects are frequently reported in intervention studies, often with a more positive effect on females than males which may warrant further investigation into the efficacy of gender specific components within interventions [96].

## 6. Limitations

While the findings indicate the potential of the SOMI-HE mental fitness intervention, several limitations are noted. Firstly, the subjective nature of wellbeing is difficult to capture [118] and self-report PA tools are vulnerable to inaccuracies and subjectivity, such as social desirability bias, and external factors, such as seasonal variation, and questionnaire complexity [119]. Nevertheless, the psychometric instruments used in this study were validated among similar populations samples and contexts. The participants provided their short-term responses to the WEMWBS, BRS, PACE+ and PASCQ scales at only two time points, as intervention programmes are often limited to two quantitative data collection time points [120]. Limited resources and access to groups of students restricted the opportunity to achieve and measure repeated outcomes, despite multiple attempts over two years. For this same reason, the ‘dose’ of the intervention was small; however, interventions can range from a short one-day programme [23] to eight weeks [37]. Bolier et al., (2013) maintained that longer interventions have greater outcomes [37]. Therefore, if permissible in the future, the exposure time to the SOMI-HE intervention components would be extended.

Gender distribution was weighted heavily by a majority female cohort; this is typical of gender disproportion in the study and the general profession of teaching in Ireland. Finally, there was no control group accessible in the study. A control group allows a researcher to draw a conclusion that any change that is identified in the intervention group is due to the exposure to the intervention being studied, rather than other factors unrelated to the study [121] Future research design will incorporate randomized control groups, a third point of data collection, and additional scales measuring processes of resilience or mental fitness, as described by Robinson et al., (2016) [17].

## 7. Conclusions

Despite these limitations, this study makes an important contribution to positive mental health promotion programme planning and implementation research. The planning process known as intervention mapping, as described in O’ Brien et al., (2020) [55], resulted in a novel and extensive mental fitness promotion intervention, that seeks to decrease stigma, increase wellbeing, peer connection, resilience skills, mental health literacy, PA levels and motivational readiness for PA change. The findings indicate that some intervention components, such as help-seeking, require augmentation. The current positive mental health intervention research process intends to take a cyclical approach to future research design and programme development. The findings of the current research will inform future programme adaptions and evaluation methods.

Promoting positive mental health requires senior ‘buy-in’ and direction [2]. Dissemination of such a programme alone depends on the layers of the social and environment, which relies on the approval of institution leadership; or individuals who ‘champion’ research in positive mental health for communities [55]. Effective health promotion programmes will have little impact if they are never used. Therefore, in a situation where an organisation is in a position to adopt the programme, a systematic plan of implementation would be essential for sustained and impactful adoption [56]. Successful dissemination of such interventions that reach the ‘real-world’ setting require implementation frameworks, such as RE-AIM (Reach, Effectiveness—Adoption, Implementation, Maintenance) [56,122]. Continued research will take such steps, as outlined comprehensively by IM [56].

## Figures and Tables

**Table 1:** State of Mind Ireland-Higher Education (SOMI-HE) intervention components, sequence, and scope.

**Day 1** **Workshop A—Positive Mental Health**
**PART 1** Understanding positive mental health—Everyone has mental health Mental fitness: stress, resilience, and vulnerability Mental health, emerging adults, and ‘one good adult’ Neuroplasticity—mindfulness and positive affirmations
**PART 2** Exercise has been known to cause health and happiness The five ways to wellbeing The transtheoretical model of change SMART—don’t find the time, make time
**Day 2** **Workshop B—Mental Health First**
**PART 1** Everyone has mental health—let’s talk stigma Mental health literacy and responding to mental health issues The stress-vulnerability bucket analogy Alcohol consumption and mental health
**PART 2** Maintaining wellbeing strategies—mindfulness Mental health literacy SMART—Resetting physical activity goals The Mental Fitness toolkit

**Table 2:** Participant demographic data.

Gender	Count	Percentage (%)
Male	39	29.1
Female	95	70.9
Age		
18–25 years	100	74.6
26–29 years	19	14.2
30 years +	15	11.2
Level of Education		
Undergraduate	19	14.2
Postgraduate	115	85.8
Year of study		
First-year	113	84.3
Second-year	17	12.7
Third-year	4	3
Type of course		
Education	100	74.6
Engineering	19	14.2
Science	13	9.7
Business	2	1.5

**Table 3:** Descriptive means for the Warwick Edinburgh Mental Well Being Scale, Brief Resilience Scale and PACE+ pre- and post-State of Mind Ireland—Higher Education intervention.

	*N*	Pre SOMI-HE Mean Score (SD)	Post SOMI-HE Mean Score (SD)	t	df	Sig (2-Tailed)
WEMWBS Male Female	124 37 87	44.52 ± 7.53 47.20 ± 7.21 43.47 ± 7.43	48.28 ± 7.52 49.23 ± 6.72 47.91 ± 7.82	−5.27	120	0.000 **
BRS Male Female	120 35 85	3.17 ± 0.68 3.47 ± 0.58 3.05 ± 0.69	3.25 ± 0.61 3.40 ± 0.60 3.19 ± 0.62	−1.75	119	0.083
PACE+ Male Female	126 37 89	2.96 ± 1.6 3.26 ± 1.54 2.84 ± 1.6	3.5 ± 1.70 4.08 ± 1.49 3.29 ± 1.73	−3.91	125	0.000 **

Note: SD = standard deviation. ** indicates *p* < 0.01.
